# Application of the INFOGEST Protocol to Evaluate the Effects of Simulated *In Vitro* Gastrointestinal Digestion on Polyphenols and Antioxidant Potential of Four Native Amazonian Fruits

**DOI:** 10.1021/acsomega.5c09934

**Published:** 2025-11-20

**Authors:** Rômulo Alves Morais, Hermanny Matos Silva Sousa, Glêndara Aparecida De Souza Martins

**Affiliations:** † Graduate Program in Food Science and Technology, Department of Food Science and Technology, Federal University of Tocantins (UFT), Palmas 77001-090, Brazil; ‡ Department of Food Science, 67739 Federal University of Lavras (UFLA), Lavras 37200-000, Brazil

## Abstract

Brazil’s native fruits, such as buriti, bacaba, guapeva, and taturubá, are rich in bioactive compounds with antioxidant and therapeutic potential. This study assessed their phenolic profile, bioaccessibility, antioxidant potential, and *in vitro* biological properties before and after simulated *in vitro* gastrointestinal digestion. The results obtained indicated that the 70% ethanolic extracts before *in vitro* digestion presented gallic acid as the major compound in the pulps of buriti, bacaba, and guapeva, with concentrations of 3284.04, 421.98, and 425.63 μg g^–1^, respectively. In contrast, the taturubá pulp revealed a higher gallic acid content in the aqueous extract (3376.48 μg g^–1^). In addition, gallic acid demonstrated high bioaccessibility, especially in the pulps of buriti (87.89%) and bacaba (77.25%). However, all the phenolic compounds tested showed significant reductions between the undigested sample and the intestinal phase (*p* < 0.05), indicating degradation processes or the creation of new unidentified compounds. Significant decreases in antioxidant potential were observed (*p* < 0.05), particularly in the oral and gastric phases: bacaba had a 95.85% reduction in DPPH inhibition (from 88.30% to 3.66%), and taturubá had a 79.10% reduction in FRAP (from 1585.51 to 331.35 mg of AAE 100 g^–1^). The reduction in antioxidant potential after digestion is relevant for functional foods, as it indicates possible degradation or modification of bioactive compounds, which can compromise their bioavailability and reduce the physiological effectiveness of antioxidant effects in the body. However, guapeva showed an 8.20% increase in antioxidant activity in the intestinal phase (from 359.57 to 389.36 mg of AAE 100 g^–1^). The fruit pulps also inhibited α-amylase activity, with the most significant inhibition in the intestinal phase: buriti (72.24%), bacaba (78.23%), guapeva (58.65%), and taturubá (47.53%). These findings suggest the potential of these fruits for the development of functional foods, although the evidence is based on *in vitro* assays and should be further validated through *in vivo* studies to confirm their physiological relevance.

## Graphical abstract

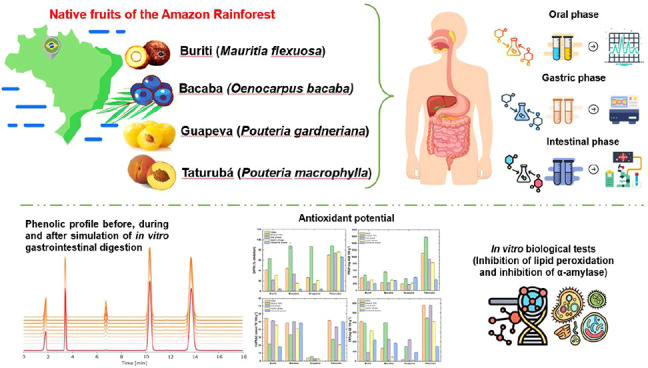

## 1 Introduction

In recent years, there has been a notable surge in the consumption of fruits and their derived products, largely attributed to their rich nutritional profiles and bioactive compounds.^1,2^ These components are recognized for their beneficial effects on human health, including their potential to mitigate the risk of mortality associated with a range of chronic and degenerative conditions. This growing trend underscores the importance of fruits as a vital component of a health-conscious diet, supported by increasing scientific evidence highlighting their role in disease prevention and overall well-being. This consumption has been associated with a reduced risk of several pathologies, especially cardiovascular and neurodegenerative diseases, and multiple types of cancer.^3,4^ In addition to their nutritional and therapeutic properties, exotic and native fruits have gained prominence due to their distinct flavor and aroma, being consumed both in natura and in various derivative products, such as juices, pulps, fermented beverages, ice creams, sweets, jams, and purees, among others.^5,6^

Brazil occupies a prominent position on the world stage as one of the main fruit producers, standing out not only for its large agricultural production but also for its great biodiversity, divided into six distinct biomes (Amazon, Cerrado, Pantanal, Caatinga, Atlantic Forest, and Pampas), housing a variety of unknown or little-explored fruits. This unique combination of agricultural productivity and ecological richness solidifies Brazil’s significance in the global agri-food sector.^7^ These native fruits have great potential as sources of bioactive compounds, mainly due to their antioxidant properties, which make them highly promising for the development of innovative and high-added-value bioproducts.^1,8,9^ Within this vast diversity, several native species have stood out for their nutritional and functional qualities, among which we can highlight four fruits: buriti (*Mauritia flexuosa*), bacaba (*Oenocarpus bacaba*), guapeva (*Pouteria gardneriana*) and taturubá (*Pouteria macrophylla*).

Buriti and bacaba are species belonging to the Arecaceae family and are predominantly found in the Amazon and Cerrado biomes of Brazil. The buriti palm, a towering plant that can grow up to 25 m in height, yields fruits characterized by their vibrant orange-red pulp. These fruits are notably rich in carotenoids, vitamin C, and unsaturated fatty acids, making them a valuable source of nutrients.^10^ On the other hand, the bacaba palm, which grows up to 15 m, produces dark-colored fruits that are famous for their high concentration of antioxidants, including polyphenols, flavonoids, and anthocyanins. In addition, bacaba fruits are an exceptional source of essential fatty acids.^1^ Both species exemplify the remarkable biodiversity and nutritional potential of Brazil’s native flora. Guapeva and taturubá, in turn, belong to the Sapotaceae family, a family that includes several tropical fruit species. Guapeva is a tree that can reach up to 20 m in height, with fruits that have thick skin and creamy, sweet pulp, rich in vitamins, especially vitamin C, and minerals.^11^ Taturubá, a tree-like plant that can also reach great heights, has fruits with brown skin and white pulp, with nutritional characteristics similar to guapeva, and these species are particularly distinguished for their antioxidant and anti-inflammatory properties, as highlighted by Silva et al.^12^ These four species exemplify the rich biodiversity of Brazilian biomes, showcasing immense nutritional value and a diverse array of bioactive compounds. These attributes make them highly sought after for applications across multiple industries, including food production, cosmetics, and pharmaceuticals, underscoring their economic and functional relevance.

The polyphenols in native fruits have attracted increasing interest due to their therapeutic potential and health benefits, especially in disease prevention.^3^ On the other hand, the bioavailability of polyphenols is notably limited, as the majority of these molecules are present in conjugated or bound forms that require prior hydrolysis by intestinal enzymes or the gut microbiota to enable absorption. Estimates indicate that merely about 5–10% of ingested polyphenols are effectively absorbed in the small intestine, with the remaining fraction undergoing extensive biotransformation in the large intestine.^13–15^ Furthermore, dietary polyphenols may play a role in modulating postprandial glycemia by inhibiting α-amylase and offering an alternative for treating type II diabetes.^16^ However, the bioavailability and bioaccessibility of these compounds, which depend on the food matrix and digestive conditions, can influence their efficacy since digestion can transform polyphenols into compounds with lower bioactivity.^17,18^ *In vitro* digestion studies are frequently used to evaluate the bioaccessibility of polyphenols in fruits and derivatives. However, data on the bioavailability and bioaccessibility of phytochemicals in native fruits remain scarce or nonexistent. Therefore, to the best of our knowledge, this is the first study to report the process of simulating *in vitro* gastrointestinal digestion in four native fruits from Brazil. Thus, this study aimed to simulate *in vitro* gastrointestinal digestion of the pulps of buriti, bacaba, guapeva, and taturubá and evaluate the bioaccessibility of individual phenolic compounds, antioxidant potential, and the inhibition of lipid peroxidation and α-amylase, as indicators of the potential antibiotic effects of these native pulps.

## 2 Materials and Methods

### 2.1 Chemical Reagents

The reagents α-amylase (30 U mg^–1^), pepsin (535 U mg^–1^), pancreatin (activity equivalent to 8 × USP), and bile salts, such as sodium glycodeoxycholate (0.8 mM), sodium taurodeoxycholate hydrate (0.45 mM), and sodium taurocholate hydrate (0.75 mM), in addition to Tripyridyl triazine (TPTZ), 2,2-diphenyl-1-picrylhydrazyl (DPPH•), 2,2′-azino-bis­(3-ethylbenzenothiazoline-6-sulfonic acid (ABTS), 6-hydroxy-2,5,7,8-tetramethylchroman-2-carboxylic acid (Trolox), thiobarbituric acid, quercetin, ferric chloride, neocuproine, potassium sulfate, ascorbic acid, sodium carbonate, molybdate sodium, aluminum chloride, ethanol, sodium phosphate, copper sulfate, and isobutanol were all purchased from Sigma-Aldrich (São Paulo, Brazil). All chemical reagents were of analytical grade.

### 2.2 Samples

Buriti (*Mauritia flexuosa*) Figure 1A and bacaba (*Oenocarpus bacaba*) Figure 1B fruits were obtained in a flooded area rich in fauna and flora in the city of Palmas, Tocantins, Brazil (Latitude—10°23′74″ S, Longitude—48°34′23″ W), between August and November to December of 2023. Guapeva fruit (*Pouteria gardneriana*) Figure 1C was harvested in the Cerrado of the city of Palmas, state of Tocantins, Brazil (Latitude—10°18′43″ S, Longitude—48°33′81″ W), between August and November of 2023. Taturubá fruits (*Pouteria macrophylla*) Figure 1D were obtained in the city of Araguatins in the state of Tocantins (Latitude—5.65615, Longitude—48.1189 5°39′22″ S, 48°7′8″ W), Legal Amazon, the northern region of Brazil, from December 2022 to January 2023.

**Figure 1:**
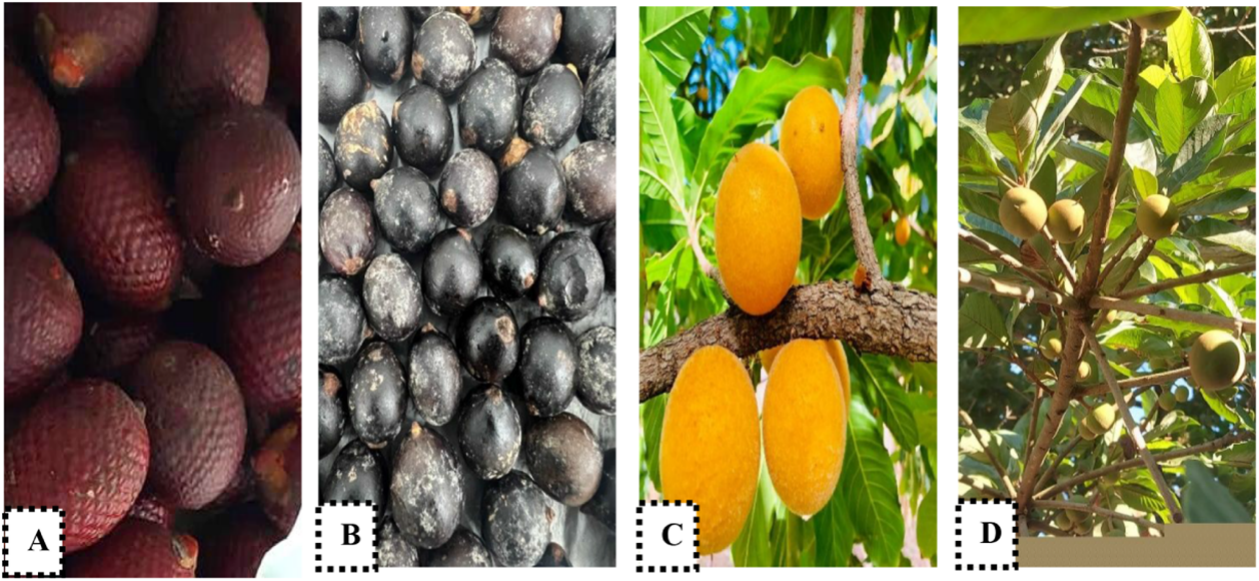
Fruits analyzed: (A) buriti (*Mauritia flexuosa*); (B) bacaba (*Oenocarpus bacaba*); (C) guapeva (*Pouteria gardneriana*); (D) taturubá (*Pouteria macrophylla*).

All fruits were harvested ripe and free from uniformity or visible physical damage (*n* = 30). Immediately after collection, the fruits were sanitized using a chlorinated solution (100 mg L^–1^) for 15 min. The pulp, peel, and seeds were manually separated with a stainless-steel knife. After collection, the edible portions from individual fruits were combined to form a composite sample for each species. This pooling approach was adopted to minimize individual variability and to obtain representative and homogeneous samples for subsequent analyses. Then, the pulps of buriti, bacaba, guapeva, and taturubá were dried at 55 °C for 24 h in a Nova Ética oven with air circulation, Model 400/4ND (São Paulo, Brazil). Then, the samples were crushed in a commercial blender until they reached a standard particle size of 1.00 mm. The different dried pulps were stored in low-density polyethylene packages and kept at −18 ± 2 °C until the analyses were performed.

### 2.3 Undigested Extract

To obtain undigested extracts of buriti, bacaba, guapeva, and taturubá pulp before *in vitro* digestion, 5 g of each sample was diluted in 50 mL of water and 70% (v/v) ethanol for comparative purposes. The extraction process was conducted using an ultrasonic bath (EGS 5HD, 40 kHz, 300 W, Enge Solutions, São Paulo, Brazil) maintained at 30 °C for 25 min. Following extraction, the samples were filtered through a membrane filter with a diameter of 13 mm and a pore size of 0.22 μm. The resulting extracts were added to amber bottles under a nitrogen atmosphere and stored at −18 °C to ensure compound stability.^19^

### 2.4 Simulation of *In Vitro* Gastrointestinal Digestion

*In vitro* simulations of gastrointestinal digestion were conducted, following the standardized protocol outlined by the COST Network consensus under FA1005 INFOGEST. This method ensures a reproducible and physiologically relevant approach to mimic the human digestive process, enabling the evaluation of bioactive compound stability and bioavailability under controlled conditions.^20,21^ This method reproduces three physiological stages of the digestion process: the oral phase, simulated with α-amylase to represent oral conditions; the gastric phase, with pepsin and HCl to simulate the stomach environment; and the intestinal phase, with bile salts, pancreatin, and sodium bicarbonate (NaHCO_3_) to mimic the conditions in the small intestine. The different fruit pulps (buriti, bacaba, guapeva, and taturubá) were analyzed separately before and after each phase of digestion (oral, gastric, and intestinal). At the end of each stage of the digestion process, the liquid fraction was collected and centrifuged at 2480 g for 10 min. The resulting supernatant was considered to be the digested sample and used to evaluate the profile of phenolic compounds and antioxidant potential. These analyses were also performed on samples collected before *in vitro* digestion for comparison.

During the oral digestion phase, 0.25 g of dried and ground pulp samples were mixed with 3.5 mL of simulated salivary fluid, 25 μL of CaCl_2_ solution, and 0.975 μL of distilled water. The pH of the mixture was adjusted to 7.0 using 0.1 M NaOH, followed by the addition of 0.5 mL of an α-amylase solution (75 U mL^–1^). The samples were incubated at 37 °C with constant agitation (100 rpm) for 2 min to simulate oral enzymatic activity. For the gastric phase, 7.5 mL of simulated gastric fluid, 20 μL of CaCl_2_, and 0.700 μL of distilled water were added to the same reaction tube. The pH was adjusted to 3.0 using 6 mol L^–1^ HCl, after which 1.6 mL of pepsin solution (2,000 U mL^–1^) was incorporated. The samples were incubated in a thermostatic bath at 37 °C for 2 h to mimic gastric digestion. In the intestinal phase, 11 mL of simulated intestinal fluid, 40 μL of CaCl_2_, 2.5 mL of bile salts, and 1.46 mL of 1 mol L^–1^ NaOH were added to the gastric digesta. The pH was adjusted to 7.0, and 5 mL of a pancreatin solution (800 U mL^–1^) was introduced. The samples were then incubated for 2 h at 37 °C under continuous agitation to reproduce intestinal conditions. At the end of each digestion stage, enzymatic activity was halted by placing the samples in an ice bath (≈4 °C). Control assays were prepared under the same conditions but without the addition of digestive enzymes or bile salts. After each digestion phase, the resulting digesta were collected for the determination of antioxidant capacity and phenolic compound profile, allowing comparison with the undigested samples. Table 1 provides a detailed description of the reagents used in the *in vitro* gastrointestinal digestion simulation including the concentrations, volumes, and compositions of enzyme and buffer solutions for each phase. This standardization ensures methodological reproducibility and accurate simulation of physiological digestive conditions.

**Table 1:** Composition and Preparation Parameters of Simulated Digestive Fluids and Enzymatic Solutions Used in the *In Vitro* Gastrointestinal Digestion Protocol^a^

		Fluids (mL)		
		Salivary	Gastric	Intestinal
Solution	Concentration	pH = 7	pH = 3	pH = 7
KCl	37.3 g L^–1^	15.1	6.9	6.8
KH_2_PO_4_	68 g L^–1^	3.7	0.9	0.8
NaHCO_3_	84 g L^–1^	6.8	12.5	42.5
NaCl	117 g L^–1^	-	11.8	9.6
MgCl_2_(H_2_O)_6_	30.5 g L^–1^	0.5	0.4	1.1
(NH4)_2_CO_3_	48 g L^–1^	0.06	0.5	-
HCl	6 mol L^–1^	0.09	1.3	0.7
H_2_O	-	373.75	365.7	338.5
NaOH	1 mol L^–1^			

		Oral phase	Gastric phase	Intestinal phase
Digestive fluid	-	3.5 mL	7.5 mL	11 mL
CaCl_2_	-	25 μL	20 μL	40 μL
H_2_O	-	0.975 μL	0.700 mL	
α-Amylase	30 U/mg	0.5 mL	-	-
Pepsin	≥250 U/mg	-	1.6 mL	-
Bile salts	-	-	-	2.5 mL
NaOH	-	-	-	1.46 mL
Pancreatin	8 × USP	-	-	5 mL

a The total volume of the fluids was standardized to 400 mL. Adapted from Minekus et al.^20^ and Morais et al.^15^

### 2.5 Preparation of Bile Salts

For the preparation of bile salts, it was necessary to obtain a mixture composed of 0.8 mM sodium glycodeoxycholate (equivalent to 0.38 g L^–1^), 0.45 mM sodium taurodeoxycholate hydrate (0.23 g L^–1^), and 0.75 mM sodium taurocholate hydrate (0.40 g L^–1^). These bile compounds were carefully combined in specific proportions to simulate the physiological conditions of the intestinal environment during the *in vitro* digestion process, ensuring the representativeness of lipid interactions and the emulsification of lipids that naturally occur in the human gastrointestinal tract.^20,21^

### 2.6 Antioxidant Potential

#### 2.6.1 DPPH• Radical Capture

The DPPH assay was performed following the procedure described by Brand-Williams, Cuvelier, and Berset.^22^ Absorbance was determined at a wavelength of 517 nm. Antioxidant activity was evaluated based on free radical scavenging capacity. The results obtained were expressed as the percentage of inhibition (% inhibition), calculated according to eq 1:

1 $$\% inhibition=\left[1-\left(\frac{AA}{AB}\right)\right]\times 100$$

Where *AA* represents the absorbance value of the sample, while *AB* represents absorbance of the control.

#### 2.6.2 Iron-Reducing Antioxidant Power (FRAP)

The determination of the antioxidant potential using the FRAP method was measured according to Benzie and Strain.^23^ The FRAP solution was prepared by combining 2.5 mL of TPTZ solution (10 mmol L^–1^) prepared in HCl medium (40 mmol L^–1^), 2.5 mL of ferric chloride hexahydrate (FeCl_3_·6H_2_O) (20 mmol L^–1^), and 25 mL of sodium acetate buffer (0.3 mol L^–1^, pH 3.6). Then, in a test tube, 90 μL of the obtained extracts, 270 μL of distilled water, and 2.7 mL of the FRAP reagent were added. The absorbance of the samples was read with a spectrophotometer at 593 nm. The results were expressed in mg of ascorbic acid equivalent per 100 g of sample (mg AAE 100 g^–1^). The calibration curve is available in the Supporting Information (Figure S1).

#### 2.6.3 Cupric Ion Reducing Antioxidant Capacity (CUPRAC)

The CUPRAC (Cupric Ion Reducing Antioxidant Capacity) assay was carried out according to the method described by Apak et al.^24^ In brief, 50 μL of the sample was added to a reaction tube containing 1 mL of copper­(II) chloride solution, 1 mL of neocuproine solution, and 1 mL of ammonium acetate buffer (pH 7.0). The mixture was incubated in the dark for 30 min at 25 °C to allow the reduction reaction to occur. After incubation, the absorbance was measured at 450 nm by using a spectrophotometer. The reducing capacity of cupric ions was quantified using a Trolox calibration curve, and the results were expressed as millimoles of Trolox equivalents per 100 g of sample (mmol TE 100 g^–1^). The calibration curve used for the calculations is provided in the Supporting Information (Figure S2).

#### 2.6.4 Total Reducing Capacity (TRC)

The total reducing capacity of hydrophilic and lipophilic compounds was assessed following the methodology described by Berker et al.^25^ Briefly, 75 μL of the Folin–Ciocalteu reagent, previously diluted 1:2 (v/v) in isobutyl alcohol, was mixed with 50 μL of the sample in a centrifuge tube. After 2 min of reaction, 875 μL of a 0.1 mol L^–1^ NaOH solution and 1.50 mL of ultrapure water were added, and the mixture was homogenized for 10 s. The tubes were then allowed to stand at room temperature and protected from light for 20 min. Subsequently, 250 μL of the reaction mixture was transferred to a microplate, and absorbance was measured at 665 nm using a spectrophotometer. The reducing capacity was expressed as milligrams of quercetin equivalents per 100 g of sample (mg QE 100 g^–1^). The calibration curve used for quantification is provided in the Supporting Information (Figure S3).

### 2.7 *In Vitro* Biological Activities

#### 2.7.1 Inhibition of Lipid Peroxidation (ILP)

The inhibition of Fe^2+^-induced lipid peroxidation in egg yolk was evaluated according to the protocol described by Daker et al.,^26^ with modifications proposed by Margraf et al.^27^ Briefly, the extracts were combined with egg yolk homogenate (pH 7.4) and incubated in a water bath at a constant temperature of 37 °C for 45 min. After incubation, a 20% acetic acid solution (pH adjusted to 3.5) and a 0.67% thiobarbituric acid (TBA) solution were added to the mixture. The reaction mixture was subsequently heated to 95 °C and maintained for 30 min. Absorbance was then measured at 517 nm by using a spectrophotometer. A control assay in which the extracts were replaced by ultrapure water was conducted under identical conditions. The percentage of lipid peroxidation inhibition was determined according to the equation described in eq 2:

2 $$\mathrm{ILP}=\frac{A_{c}-A_{a}}{A_{c}}\times 100$$

Where ILP is the inhibition of lipid peroxidation (%); *A*_c_ is the absorbance of the control sample; and *A*_a_ is the absorbance of the sample.

#### 2.7.2 Inhibition of α-Amylase

The α-amylase inhibitory activity of the extracts was determined following the methodology described by Ademiluyi and Oboh.^28^ Briefly, 50 μL of the extract was mixed with 500 μL of an α-amylase solution prepared in 20 mM sodium phosphate buffer (pH 6.9) and incubated at 25 °C for 10 min. Subsequently, 500 μL of a 1% (w/v) starch solution was added, and the mixture was incubated again at 25 °C for an additional 10 min. After incubation, 1 mL of 3,5-dinitrosalicylic acid (DNS) reagent at a concentration of 60 nM was added, and the reaction mixture was heated at 95 °C for 5 min. The samples were then rapidly cooled in an ice bath (≈4 °C), followed by the addition of 10 mL of distilled water. The absorbance of the resulting solution was measured at 540 nm by using a UV–Vis spectrophotometer. A control was prepared under identical conditions, with the extract replaced by 20 mM sodium phosphate buffer (pH 6.9). The percentage inhibition of α-amylase activity was calculated according to eq 3:

3 $$\alpha ‐amylase\;\mathrm{inhibition}=\frac{A_{c}-A_{a}}{A_{c}}\times 100$$

Where *A*_c_ is the absorbance of the control sample and *A*_a_ is the absorbance of the sample.

### 2.8 Phenolic Compounds Profile

The phenolic profiles of buriti, bacaba, guapeva, and taturubá pulp extracts, before and after each phase of *in vitro* digestion (oral, gastric, and intestinal), were analyzed by high-performance liquid chromatography (HPLC). Before injection, the extracts were filtered using a 13 mm diameter and a 0.22 μm pore membrane filter. The quantification and identification of phenolic compounds were performed in an HPLC-DAD-UV–VIS system (AB Sciex LLC, Framingham, USA), consisting of a quaternary pump LC-20AT, degasser DGU-20A5 (serial no.: L20244808404), injector SIL-20th (serial no.: L20164503197), controller CBM-20th (serial no.: L20234505269), oven CTO-20AC (serial no.: L20214503287), detector SPDM-20th (serial no.: L20154503047), detector RID-10th (serial no.: C20934806770), and fraction collector FRC-10^a^ (serial no.: C20374504580). The analysis was conducted under the following conditions: oven temperature at 35 °C, Shim-pack VP-ODS column (250 mm × 4.6 mm × 5 μm), and Shim-pack GVP-ODS precolumn (10 mm × 4.6 mm × 5 μm). The mobile phase consisted of two components: phase A was prepared as a 2% (v/v) solution of glacial acetic acid in water, and phase B, a blend of methanol, water, and acetic acid in a ratio of 70:28:2% (v/v). The system operated at a constant flow rate of 1.0 mL min^–1^, utilizing a gradient elution protocol that extended over a total duration of 65 min. The injection volume was 20 μL, and phenolic compounds were detected at 280 nm. Calibration curves were obtained in duplicate, using 10 different concentrations of each standard. The calibration curves were as follows: trigonelline (*y* = 49881*x* – 553.26, *r* ^2^ = 0.9986), theobromine (*y* = 29533*x* – 47.97, *r* ^2^ = 0,9989), catechin (*y* = 11371x – 567.84, *r* ^2^ = 0.9993), vanillin (*y* = 69247*x* + 254.33, *r* ^2^ = 0.9986), gallic acid (*y* = 3994*x* + 8047, *r* ^2^ = 0.9962), syringic acid (*y* = 49401*x* + 706.65, *r* ^2^ = 0.9990), chlorogenic acid (*y* = 29451*x* – 2224.70, *r* ^2^ = 0.9992), caffeic acid (*y* = 67928*x* – 2499.20, *r* ^2^ = 0.9991), *p*-coumaric (*y* = 100602*x* – 4097.70, *r* ^2^ = 0.9989), *o*-coumaric (*y* = 116320*x* – 6657, *r* ^2^ = 0.9990), *m*-coumaric acid (*y* = 130558x – 2496.20, *r* ^2^ = 0.9996), ferulic acid (*y* = 52609*x* – 5197.10, *r* ^2^ = 0.9997), rosmarinic acid (*y* = 30075*x* – 5006.10, *r* ^2^ = 0.9984), *trans*-cinnamic acid (*y* = 177527*x* – 8753, *r* ^2^ = 0.9989), and resveratrol (*y* = 79962*x* – 11037, *r* ^2^ = 0.9983). The phenolic compounds were identified by comparing the sample peaks’ retention times with the standards’ retention times. The results were expressed in μg g^–1^ of the sample.

#### 2.8.1 Bioaccessibility Index

The effect of *in vitro* gastrointestinal simulation on the individual phenolic profile of the samples was evaluated using the bioaccessibility index (BI), calculated according to eq 4:

4 $$\mathrm{BI}=\frac{A}{B}\times 100$$

Where *A* = total phenolic content after *in vitro* digestion and *B* = total phenolic content before *in vitro* digestion.

### 2.9 Statistical Analyses

Statistical differences among the groups were evaluated using the Tukey’s test and Student’s *t*-test to compare mean values, with significance set at 5% (*p* < 0.05). Statistical analyses were performed using Statistica 10.0 software (StatSoft Inc., Tulsa, OK, USA). To examine the relationships between bioactive compounds and antioxidant activity, Pearson’s correlation analysis was employed. Data analysis and graphical representations were generated using OriginPro 2022 software (OriginLab Corporation, Northampton, MA, USA).

## 3 Results and Discussion

### 3.1 Phenolic Profile and Simulation of *In Vitro* Gastrointestinal Digestion of Buriti Pulp

The phenolic profiles of the undigested (70% v/v ethanol and water) and digested extracts of buriti pulp are presented in Table 2. This is the first study to evaluate the effects of simulated *in vitro* gastrointestinal digestion on the individual phenolic compounds of four native Brazilian fruits. Of the 15 phenolic compounds investigated, only 11 were detected and quantified in buriti pulp. Two main groups were identified and quantified: flavonols and phenolic acids. Alkaloids were also quantified but in smaller amounts. The undigested extracts obtained with water and 70% ethanol presented gallic acid (736.10 and 3284.01 μg g^–1^) as the main compounds, followed by catechin (35.01 and 40.80 μg g^–1^, respectively). On the other hand, Silva et al.^29^ reported significantly lower concentrations for the same compounds, reporting values of 0.062 and 961.21 μg g^–1^, respectively. Such variations may arise from factors including the stage of fruit maturation, exposure to sunlight, duration of storage, prevailing climatic conditions, soil mineral composition, and water availability.^19^ Furthermore, inconsistencies in analytical methodologies, as well as differences in the selectivity and accuracy of the instruments employed, may also contribute to the observed discrepancies in the results.

**Table 2:** Phenolics Profile before and after Simulated *In Vitro* Digestion of Buriti Pulp and Bioaccessibility Index[Table-fn tbl2fn1]

Buriti pulp (*Mauritia flexuosa*)								
Individual phenolics (μg g^–1^)	RT	Nondigested H_2_O	Nondigested ethanol 70%	Oral phase	Gastric phase	Intestinal phase	Bioaccessibility (%) H_2_O	Bioaccessibility (%) ethanol 70%
Trigonelline	3.49	14.25 ± 0.12^b^	30.50 ± 1.28^a^	11.40 ± 0.56^c^	10.97 ± 0.13^c^	11.56 ± 0.22^c^	81.12	37.90
Gallic acid	6.65	1010.91 ± 0.71^b^	3284.01 ± 4.52^a^	170.19 ± 0.95^d^	146.16 ± 0.47^e^	888.58 ± 1.16^c^	87.89	27.05
Theobromine	9.30	nd.	49.97 ± 1.43^a^	28.02 ± 0.05^c^	29.23 ± 0.38^b^	nd	–	–
Catechin	10.49	35.01 ± 0.86^b^	40.80 ± 0.61^a^	13.32 ± 0.17^c^	33.15 ± 1.20^b^	13.82 ± 0.52^c^	39.48	33.87
Chlorogenic acid	12.19	2.52 ± 0.40^c^	16.05 ± 1.44^a^	9.78 ± 0.09^b^	9.57 ± 0.23^b^	nd	–	–
Caffeic acid	14.38	6.14 ± 0.01^b^	7.06 ± 0.11^a^	nd	nd	nd	–	–
Syringic acid	15.39	nd	nd	nd	nd	nd	–	–
Vanillin	16.80	5.39 ± 0.02	nd	nd	nd	nd	–	–
*p*-Coumaric acid	20.65	7.13 ± 0.89^a^	8.29 ± 0.70^a^	nd	nd	nd	–	–
Ferulic acid	23.57	24.60 ± 0.63^a^	nd	14.45 ± 0.18^c^	22.12 ± 0.61^a^	16.51 ± 0.02^b^	67.11	–
*m*-Coumaric acid	25.95	1.46 ± 0.05	nd	nd	nd	nd	–	–
*o*-Coumaric acid	32.35	nd	2.72 ± 0.22	nd	nd	nd	–	–
Resveratrol	36.73	nd	nd	nd	nd	nd	–	–
Rosmarinic acid	42.83	nd	nd	nd	nd	nd	–	–
*trans*-Cinnamic acid	50.43	nd	nd	nd	nd	nd	–	–

i Results are presented as mean ± standard deviation (*n* = 2); means followed by the same lowercase letter in the same row do not differ statistically using the Tukey’s test or *t*-test at 5% probability (*p* ≤ 0.05); nd: not detected; RT: retention time in minutes.

Therefore, gallic acid is a secondary metabolite found in a wide variety of plants, vegetables, nuts, and fruits, possessing excellent anti-inflammatory and antioxidant activities, in addition to acting as an antimicrobial and helping to modulate the diversity of the intestinal flora, being widely used to treat diseases such as diarrhea, dysentery, and internal bleeding, as well as to reduce inflammation and other gastrointestinal diseases.^30–32^ In accordance with the functional claims reported for gallic acid, catechin and its derivatives have been shown to present abundant pharmacological effects, including antihypertensive and antiviral activity, reduction of cardiovascular diseases, anticancer properties, reduction of plasma cholesterol levels, protection against neurodegenerative diseases, among other therapeutic and functional claims.^33,34^ Thus, the substantial levels of gallic acid and catechin identified in buriti pulp highlight its remarkable therapeutic, functional, and technological potential. This fruit emerges as a significant reservoir of bioactive compounds, exhibiting antioxidant, anti-inflammatory, and antimicrobial properties.^29^ These attributes position buriti as a promising candidate for the development of novel food and pharmaceutical applications aimed at enhancing health and overall well-being.

Although 11 compounds (trigonelline, theobromine, catechin, vanillin, gallic acid, chlorogenic acid, Syringic acid, caffeic acid, *p*-coumaric acid, *o*-coumaric acid, and ferulic acid) were quantified in the undigested extracts, only four compounds (trigonelline, gallic acid, ferulic acid, and catechin) were identified and quantified in the final stage of digestion (intestinal phase). This indicates significant degradation during *in vitro* simulated gastrointestinal digestion. In general, gallic acid exhibited the highest bioaccessibility among the phenolics for water and 70% ethanol extracts, with values of 87.89% and 27.05%, respectively. Phenolic acids with low molecular weight, such as gallic acid, are efficiently absorbed in the gastrointestinal tract, contributing to their elevated bioaccessibility.^35,36^ These findings align with those of Dutra et al.,^13^ Morais et al.,^15^ and Berni et al.,^37^ who observed minimal reductions in gallic acid concentrations after gastrointestinal digestion of siriguela (*Spondias purpurea*) and buriti (*Mauritia flexuosa*), respectively. In contrast, catechin demonstrated a lower bioaccessibility, with 60.52% and 66.12% reductions between the undigested sample and the intestinal phase for water and 70% ethanol, respectively. Given the widespread occurrence of phenolic compounds, the stability and bioaccessibility of these substances are influenced by their interactions with the food matrix, which may explain the variability observed across different plant-based foods.^38^

### 3.2 Phenolic Profile and Simulation of *In Vitro* Gastrointestinal Digestion of Bacaba Pulp

The phenolic composition of bacaba pulp, both before and after *in vitro* digestion, is shown in Table 3. Statistical analysis revealed significant differences (*p* < 0.05) across all samples, highlighting substantial variations in phenolic content throughout the digestive process. Among the identified compounds in the aqueous and ethanolic extracts of bacaba pulp, gallic acid (196.75 and 421.98 μg g^–1^, respectively) and chlorogenic acid (135.99 and 136.60 μg g^–1^, respectively) were predominant. These compounds have been extensively researched for their antioxidant capacity and potential health benefits. Gallic acid, in particular, is recognized for its strong antioxidant, anti-inflammatory, and anticancer properties.^39–41^ These findings suggest that regular intake of gallic acid may contribute to a reduced risk of cardiovascular diseases and cancer, as well as enhanced cognitive function. Similarly, chlorogenic acid, a major phenolic compound found in various fruits and vegetables, has been linked to improved glucose metabolism and reduced blood pressure, with evidence indicating its role in lowering the risk of type 2 diabetes.^42,43^ These results underscore the value of incorporating foods rich in these bioactive compounds into the diet for health promotion and chronic disease prevention.

**Table 3:** Phenolics Profile before and after Simulated *In Vitro* Digestion of Bacaba Pulp and the Bioaccessibility Index[Table-fn tbl3fn1]

Bacaba pulp (*Oenocarpus bacaba*)								
Individual phenolics (μg g^–1^)	RT	Nondigested H_2_O	Nondigested ethanol 70%	Oral phase	Gastric phase	Intestinal phase	Bioaccessibility (%) H_2_O	Bioaccessibility (%) ethanol 70%
Trigonelline	3.49	93.50 ± 0.54^a^	49.17 ± 0.23^b^	31.54 ± 0.98^c^	33.26 ± 0.81^c^	22.09 ± 0.13^d^	23.63	44.93
Gallic acid	6.65	196.75 ± 0.26^b^	421.98 ± 1.50^a^	39.89 ± 1.20^e^	68.47 ± 0.11^d^	152.01 ± 1.35^c^	77.25	36.02
Theobromine	9.30	114.25 ± 0.53^b^	134.36 ± 0.94^a^	56.48 ± 0.53^c^	4.92 ± 0.57^d^	nd	–	
Catechin	10.49	36.84 ± 0.51^a^	24.08 ± 0.28^b^	6.84 ± 0.44^d^	14.70 ± 0.05^c^	nd	–	–
Chlorogenic acid	12.19	135.99 ± 1.29^a^	136.60 ± 0.05^a^	3.96 ± 0.61^c^	66.45 ± 0.03^b^	nd	–	–
Caffeic acid	14.38	32.72 ± 0.21^b^	35.93 ± 0.04^a^	3.84 ± 0.50^e^	26.12 ± 0.20^c^	8.42 ± 0.07^d^	25.74	23.44
Syringic acid	15.39	31.44 ± 1.49^a^	17.08 ± 0.56^c^	9.66 ± 0.34^d^	25.31 ± 0.05^b^	7.53 ± 0.55^e^	23.95	44.08
Vanillin	16.80	1.93 ± 0.05^b^	4.93 ± 0.07^a^	nd	nd	nd	–	–
*p*-Coumaric acid	20.65	17.50 ± 0.80^a^	19.93 ± 0.87^a^	2.57 ± 0.30^d^	10.34 ± 0.65^b^	5.45 ± 0.23^c^	31.14	27.35
Ferulic acid	23.57	18.26 ± 0.06^a^	15.50 ± 0.30^b^	4.82 ± 0.93^c^	15.21 ± 0.07^b^	5.39 ± 0.19^c^	29.52	34.77
*m*-Coumaric acid	25.95	1.52 ± 0.05^b^	5.40 ± 0.10^a^	nd	nd	nd	–	–
*o*-Coumaric acid	32.35	nd	nd	nd	nd	nd	–	–
Resveratrol	36.73	nd	2.30 ± 0.01	nd	nd	nd	–	–
Rosmarinic acid	42.83	nd	nd	nd	nd	nd	–	–
*trans*-Cinnamic acid	50.43	nd	nd	nd	nd	n	–	–

i Results are presented as mean ± standard deviation (*n* = 2); means followed by the same lowercase letter in the same row do not differ statistically using the Tukey’s test or *t*-test at 5% probability (*p* ≤ 0.05); nd: not detected; RT: retention time in minutes.

Following the simulation of gastrointestinal digestion, a reduction in phenolic content was observed across all compounds compared with their undigested forms. This decline is attributed to the extensive metabolism of polyphenols during digestion, where processes such as hydrolysis and oxidation transform them into metabolites distinct from their original structures. The digestive environment, influenced by dietary components like proteins and lipids, as well as factors such as enzymatic activity, pH fluctuations, and bile salts, can alter the chemical structure, solubility, and bioavailability of these compounds.^20,21^ Consequently, during *in vitro* digestion and fermentation, higher molecular weight phenolics may degrade, yielding low molecular weight derivatives and altering the bioaccessibility of polyphenolic compounds.

Thus, the bioaccessibility of phenolic compounds is a crucial factor in determining their potential physiological effects after food ingestion. In the present study, the bioaccessibility of gallic acid was observed at 77.25% for the aqueous extract and 36.02% for the ethanolic extract, indicating a considerably higher bioaccessibility in the aqueous extract. These values, although significant, reflect moderate and low bioaccessibility, respectively, which suggests that despite the high concentrations of gallic acid quantified in the undigested extracts, the absorption and utilization of this compound in the gastrointestinal tract are limited. This behavior can be attributed to the interaction of phenolic compounds with food components, which can hinder their release and absorption in the intestine. Recent studies indicate that, although gallic acid is widely found in various foods, its availability for absorption by the body can be reduced due to the formation of complexes with other substances present in the food.^36,44^

Furthermore, the phenolic compounds present in bacaba pulp generally exhibited bioaccessibility of ≤45%, with values varying according to the type of extract. Although these compounds are present at relatively high concentrations in the raw material, such limited bioaccessibility may constrain their potential biological impact. This pattern aligns with recent literature, which highlights that the chemical structure of phenolics and the physical and biochemical barriers of the gastrointestinal tract often restrict their absorption. Despite this limitation, these compounds retain significant bioactivity, as evidenced by their antioxidant and enzyme-inhibitory effects reported in recent studies, suggesting that even partially bioaccessible phenolics can contribute to functional effects.^45,46^ The vast majority of these phenolic compounds are associated with complex polymeric structures or are found in forms that hinder their release during digestion. This aspect is particularly significant when evaluating the intake of plant-based extracts or natural foods abundant in phenolic compounds. While these substances exhibit strong antioxidant and anti-inflammatory effects, their biological effectiveness is heavily influenced by their capacity to be absorbed and metabolized within the human body.^3,47^

### 3.3 Phenolic Profile and Simulation of *In Vitro* Gastrointestinal Digestion of Guapeva Pulp

The phenolic profile values before and during the *in vitro* digestion phases of guapeva pulp are presented in Table 4. The results indicated significant differences between the samples (*p* < 0.05), evidencing substantial variations in the phenolic compounds during the digestive process. The main compounds identified in the guapeva pulp were gallic acid and caffeic acid, with concentrations ranging from 52.54 to 425.63 μg g^–1^ for gallic acid and from 10.91 to 87.78 μg g^–1^ for caffeic acid. After simulating the *in vitro* digestion of the samples, the phenolic compounds presented nonuniform behavior. However, most of the individual phenolics tested showed a decreasing trend between the phases: before digestion > oral phase > gastric phase > intestinal phase. Studies have shown that the bioaccessibility of phenolic compounds can increase during gastric digestion. In contrast, intestinal digestion promotes reductions in the concentrations of different polyphenols, probably related to their stability, structural changes, and degradation.^44,48,49^ Caffeic acid, a metabolite derived from chlorogenic acid, is widely recognized for its antioxidant and anti-inflammatory properties. It has also been linked to enhanced liver function and a decreased risk of metabolic disorders, including type 2 diabetes and obesity.^50,51^ Studies indicate that the regular consumption of foods rich in these phenolic compounds can aid in regulating blood glucose levels, improving cardiovascular health, and exerting neuroprotective effects. These benefits may contribute to a reduced risk of neurodegenerative conditions, such as Alzheimer’s and Parkinson’s diseases.^1,17^ These data reinforce the importance of incorporating natural sources of these compounds into the diet, given their potential to promote health and prevent chronic diseases.

**Table 4:** Phenolics Profile before and after Simulated *In Vitro* Digestion of Guapeva Pulp and Bioaccessibility Index[Table-fn tbl4fn1]

Guapeva pulp (*Pouteria gardneriana*)								
Individual phenolics (μg g^–1^)	RT	Nondigested H_2_O	Nondigested ethanol 70%	Oral phase	Gastric phase	Intestinal phase	Bioaccessibility (%) H_2_O	Bioaccessibility (%) ethanol 70%
Trigonelline	3.49	57.12 ± 0.37^b^	65.67 ± 0.46^a^	6.90 ± 0.27^c^	1.91 ± 0.02^d^	7.25 ± 1.30^c^	12.69	86.98
Gallic acid	6.65	234.43 ± 0.38^b^	425.63 ± 0.30^a^	52.54 ± 0.68^e^	161.42 ± 0.88^d^	180.42 ± 0.22^c^	76.96	55.07
Theobromine	9.30	3.48 ± 0.26^c^	19.64 ± 0.54^a^	0.52 ± 0.02^e^	10.61 ± 0.23^b^	1.17 ± 0.47^d^	33.62	17.71
Catechin	10.49	31.84 ± 0.99^a^	23.98 ± 0.72^b^	5.32 ± 0.35^d^	4.99 ± 0.45^d^	11.23 ± 1.65^c^	35.27	46.83
Chlorogenic acid	12.19	30.84 ± 0.18^b^	77.05 ± 0.10^a^	7.95 ± 0.20^e^	23.88 ± 0.16^c^	10.61 ± 0.36^d^	34.45	40.03
Caffeic acid	14.38	61.80 ± 0.24^b^	87.78 ± 0.98^a^	34.43 ± 0.36^c^	11.25 ± 0.27^d^	10.94 ± 0.43^d^	17.70	12.46
Syringic acid	15.39	5.06 ± 0.05^b^	19.97 ± 1.26^a^	2.52 ± 0.17^c^	1.78 ± 0.03^d^	nd	–	–
Vanillin	16.80	nd	11.87 ± 1.12^a^	2.09 ± 0.01^d^	7.61 ± 0.12^b^	5.10 ± 0.03^c^	–	42.97
*p*-Coumaric acid	20.65	3.66 ± 0.31^b^	19.74 ± 0.95^a^	0.74 ± 0.18^c^	nd	nd	–	–
Ferulic acid	23.57	nd	15.85 ± 0.32^a^	3.53 ± 0.17^b^	2.12 ± 0.04^c^	nd	–	–
*m*-Coumaric acid	25.95	nd	5.99 ± 0.17	nd	nd	nd	–	–
*o*-Coumaric acid	32.35	nd	nd	nd	nd	nd	–	–
Resveratrol	36.73	nd	nd	nd	nd	nd	–	–
Rosmarinic acid	42.83	nd	nd	nd	nd	nd	–	–
*trans*-Cinnamic acid	50.43	nd	0.81 ± 0.02	nd	nd	nd	–	–

i Results are presented as mean ± standard deviation (*n* = 2); means followed by the same lowercase letter in the same row do not differ statistically using the Tukey’s test or *t*-test at 5% probability (*p* ≤ 0.05); nd: not detected; RT: retention time in minutes.

### 3.4 Phenolic Profile and Simulation of *In Vitro* Gastrointestinal Digestion of Taturubá Pulp

The phenolic profile values before and during the *in vitro* digestion phases of Taturubá pulp are presented in Table 5. The main compounds identified in taturubá pulp were chlorogenic acid, syringic acid, and ferulic acid, whose concentrations varied considerably during digestion. In general, the bioaccessibility of these compounds was influenced by the solvent used in the extraction before the *in vitro* gastrointestinal digestion simulation process (*p* < 0.05). Thus, it was observed that, for Taturubá pulp, the most efficient solvent to promote the release of phenolic compounds was 70% alcohol, while water was inefficient in making the compounds bioaccessible. This result is in line with the literature, which indicates that organic solvents, such as ethanol and methanol, tend to be more effective in extracting phenolic compounds since these solvents can better dissolve lipophilic compounds present in plant matrices.^52^

**Table 5:** Phenolics Profile before and after Simulated *In Vitro* Digestion of Taturubá Pulp and the Bioaccessibility Index[Table-fn tbl5fn1]

Taturubá pulp (*Pouteria macrophylla*)								
Individual phenolics (μg g^–1^)	RT	Nondigested H_2_O	Nondigested ethanol 70%	Oral phase	Gastric phase	Intestinal phase	Bioaccessibility (%) H_2_O	Bioaccessibility (%) ethanol 70%
Trigonelline	3.49	30.13 ± 0.91^a^	20.07 ± 0.78^b^	20.50 ± 0.69^b^	10.39 ± 0.02^c^	10.26 ± 0.24^c^	34.05	51.12
Gallic acid	6.65	3376.48 ± 2.44^a^	1863.07 ± 0.41^c^	2776.24 ± 5.14^b^	1371.43 ± 1.63^d^	1371.48 ± 0.75^d^	40.62	73.61
Theobromine	9.30	nd	nd	nd	nd	nd	–	–
Catechin	10.49	20.36 ± 0.66^a^	8.04 ± 0.08^b^	7.13 ± 0.09^c^	2.18 ± 0.09^e^	6.08 ± 0.06^d^	29.86	75.62
Chlorogenic acid	12.19	163.84 ± 1.33^a^	15.93 ± 0.64^b^	1.06 ± 0.02^d^	nd	2.48 ± 0.48^c^	1.51	15.56
Caffeic acid	14.38	nd	0.66 ± 0.02	nd	nd	nd	–	–
Syringic acid	15.39	nd	751.04 ± 10.63^a^	605.13 ± 5.43^b^	79.59 ± 0.98^c^	50.06 ± 0.61^d^	–	6.66
Vanillin	16.80	158.87 ± 1.28	nd	nd	nd	nd	–	–
*p*-coumaric acid	20.65	5.12 ± 0.02^a^	nd	2.50 ± 0.02^b^	0.59 ± 0.01^c^	1.15 ± 0.31^c^	22.46	–
Ferulic acid	23.57	99.60 ± 0.74^c^	211.49 ± 1.70^a^	110.70 ± 0.31^b^	83.21 ± 1.04^d^	42.69 ± 0.33^e^	42.86	20.18
*m*-coumaric acid	25.95	nd	0.32 ± 0.02	nd	nd	nd	–	–
*o*-coumaric acid	32.35	nd	1.54 ± 0.06	nd	nd	nd	–	–
Resveratrol	36.73	nd	nd	nd	nd	nd	–	–
Rosmarinic acid	42.83	nd	nd	nd	nd	nd	–	–
*trans*-cinnamic acid	50.43	nd	28.12 ± 0.03^a^	21.01 ± 1.28^b^	5.84 ± 0.06^c^	5.74 ± 0.37^c^	–	20.41

i Results are presented as mean ± standard deviation (*n* = 2); means followed by the same lowercase letter in the same row do not differ statistically using the Tukey’s test or *t*-test at 5% probability (*p* ≤ 0.05); nd: not detected; RT: retention time in minutes.

Catechin was not the predominant phenolic compound in the samples; however, it showed the highest bioaccessibility (75.62%) when compared to the other compounds, followed by gallic acid (73.61%). Catechin is a flavonoid widely present in foods of plant origin, such as green tea, and it has been associated with several health benefits. Recent studies demonstrate that catechin exerts significant antioxidant and anti-inflammatory effects, contributing to reducing the risk of cardiovascular disease, improving endothelial health, and reducing oxidized LDL levels.^34,53^ When combined with other phenolic compounds, such as ferulic acid and chlorogenic acid, catechin can amplify its antioxidant and anti-inflammatory effects. Ferulic acid, for example, has been associated with protection against DNA damage and oxidative stress, which are important factors in the developent of chronic diseases such as cancer.^54^ Chlorogenic acid, in turn, has been widely studied for its antidiabetic and cardioprotective properties, in addition to demonstrating potential in modulating lipid metabolism and reducing blood pressure.^55^ The synergistic interaction of catechin, ferulic acid, and chlorogenic acid, which are bioactive compounds found in Taturubá pulp, may significantly enhance cardiovascular health, improve glycemic regulation, and provide robust protection against inflammatory pathways. This unique combination underscores the substantial functional potential of these compounds, making them promising candidates for the formulation of innovative food products and dietary supplements with pronounced bioactive benefits.^34,53,54^

The evaluation of the bioaccessibility of phenolic compounds present in the pulps of different fruits revealed distinct behaviors among the samples, depending on the solvent used for extraction. Buriti pulp presented the best bioaccessibility when extracted with water, indicating that the phenolic compounds of this plant matrix are more easily released in the aqueous form. This result can be explained by the water-soluble nature of some of the predominant phenolic compounds in buriti pulp, such as phenolic acids, which are known to have greater solubility in polar solvents, such as water.^1,56^ This behavior of buriti pulp reflects a pattern similar to that observed for bacaba and guapeva pulp, which also presented greater bioaccessibility when extracted with water. Therefore, water appears to be more effective for extracting and releasing phenolic compounds in these samples, possibly due to the presence of water-soluble compounds with greater affinity for polar solvents.^57,58^

On the Taturubá pulp, an opposite behavior was observed, with greater bioaccessibility of phenolic compounds when extracted with 70% ethanol. This result suggests that in the Taturubá pulp, the predominant phenolic compounds have a greater affinity for organic solvents, such as ethanol, which may be related to the presence of lipophilic phenolic compounds or the interaction between these compounds and nonpolar components of the food matrix. Ethanol is recognized for its ability to dissolve a broad spectrum of phenolic compounds, such as flavonoids and phenolic acids, which typically display enhanced solubility in solvents with lower polarity.^20,52,57^ The difference in behavior among the buriti, bacaba, guapeva, and Taturubá pulps can be attributed to the different proportions and chemical characteristics of the phenolic compounds present in each fruit, which may vary according to the type of fruit, cultivation conditions, and processing method. These variations in the phenolic profile and bioaccessibility of the pulps investigated highlight the importance of understanding the interaction between phenolic compounds and the solvents used for extraction, in addition to providing insights into the differences in the biochemical composition of each fruit.^18,59^ The observed pattern indicates that to maximize the bioaccessibility of phenolic compounds and unlock their potential health benefits, it is crucial to choose the appropriate solvent tailored to each specific fruit type. Thus, understanding this extraction and bioaccessibility variable is crucial for developing functional foods that maximize the health benefits associated with the consumption of these bioactive compounds.

### 3.5 Antioxidant Potential before and after Simulation of *In Vitro* Gastrointestinal Digestion

The antioxidant capacity of undigested fruits, as well as during the oral, gastric, and intestinal phases of a simulated *in vitro* gastrointestinal digestion process, was assessed through four distinct antioxidant assays (DPPH, FRAP, CUPRAC, and total reducing capacity (TRC)), both prior to and during simulated digestion (Figure 2). Evaluating each proposed method separately, it was observed that the antioxidant potential estimated by the DPPH radical scavenging method was significantly reduced between the undigested samples and after the digestive process for all fruits analyzed (*p* ≤ 0.05). The best results for the DPPH assay in the undigested samples in the ethanolic extract were 88.30, 87.65, and 88.53% inhibition for bacaba, guapeva, and taturubá, respectively, with no significant differences between them (*p* ≤ 0.05). On the other hand, buriti pulp presented lower results (64.12% inhibition). However, at the end of the digestion process, bacaba, guapeva, and taturubá had reductions of 95.85% (from 88.30 to 3.66% inhibition), 95.58% (from 87.65 to 3.88% inhibition), and 27.78% (from 88.53 to 66.59% inhibition) (Figure 2). The results align with those reported by de Paulo Farias et al.^60^ for uvaia (*Eugenia pyriformis*) and Schulz et al.^61^ for jussara (*Euterpe edulis*), where a marked decrease in antioxidant activity, assessed using the DPPH assay, was observed in both fractions of the fruits subjected to simulated *in vitro* digestion.

**Figure 2:**
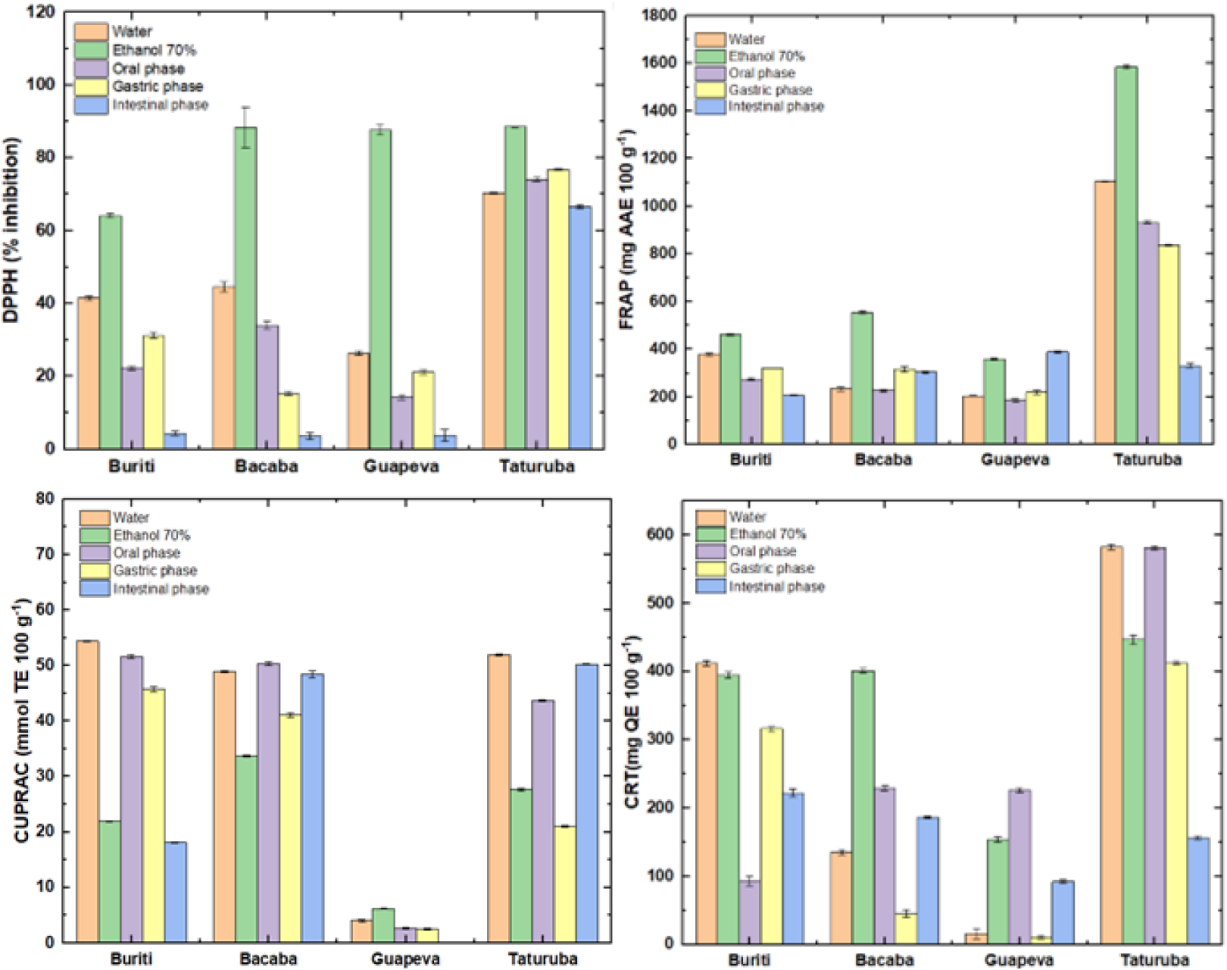
Antioxidant potential of buriti, bacaba, guapeva, and taturubá before and after *in vitro* digestion phases (oral, gastric, and intestinal).

Using the ferric ion reduction anaphylaxis (FRAP) method, the undigested fractions in the ethanol extract of buriti, bacaba, and taturubá pulp showed the highest antioxidant potential of 461.67, 554.74, and 1585.51 mg of AAE 100 g^–1^, respectively. However, the results of this method decreased drastically after the end of the *in vitro* digestion simulation process, showing a reduction of 55.30% (206.35 mg AAE 100 g^–1^) for buriti, 63.09% (204.74 mg AAE 100 g^–1^) for bacaba, and 79.10% (331.35 mg AAE 100 g^–1^) for taturubá (Figure 2). On the other hand, it was observed that the antioxidant potential of guapeva was considerably improved after the simulated *in vitro* digestion process, with an increase of approximately 8.20% between the undigested sample and the intestinal phase (from 359.57 to 389.36 mg AAE 100 g^–1^). This behavior was the same as observed by Chen et al.^62^ when studying the antioxidant capacity of 33 fruits subjected to simulated *in vitro* digestion; the authors found that after the digestive process, the antioxidant potential by the FRAP method increased after the intestinal phase in fruits such as banana, grape, nectarine, and peach.

The differences observed in the DPPH and FRAP assays between undigested and digested samples (intestinal phase) during simulated *in vitro* digestion can be ascribed to various factors influencing the bioavailability of polyphenols and, as a result, the stability of the antioxidant potential during digestion, such as different pH conditions that influence the proportion of phenolic compounds, leading to their degradation or transformation, resulting in new complex phenolics with various chemical properties, bioavailability, and biological activities, which can render individual phenolic compounds detectable or undetectable, among other factors.^14,63–65^

For the CUPRAC and TCR assays, the antioxidant potential behaved differently depending on the fruit studied (Figure 3). However, for both methods, all fruits showed significant drops in their results when comparing the undigested ethanolic extract with the intestinal phase (*p* ≤ 0.05). For CUPRAC, the most significant drop observed was in the buriti pulp (from 54.40 to 18.15 mmol of TE g^–1^), followed by the guapeva pulp (from 6.17 to 0.30 mmol of TE g^–1^). Regarding the TCR analysis, the smallest drops were reported for the bacaba pulp (from 401.28 to 186.15 mmol of TE g^–1^) and for the taturubá pulp (from 582.65 to 156.40 mmol of TE g^–1^). According to Giusti et al.^66^ and Ketnawa et al.,^64^ these variations between stages of *in vitro* digestion occur because phenolics can be released from the matrix in limited amounts during digestion. Thus, the physical interaction between free phenolics and cell wall material results in low bioavailable compounds for absorption in the intestinal part, making them more accessible in the gastric phase. In addition, covalent bonds between cell wall polysaccharides and phenolic acids (mainly ferulic acid) prevent related phenolics from being cleaved by human pancreatic enzymes, which is consistent with the results observed in the samples studied here, which present significant amounts of ferulic acid in their composition.^17,20,21,46,60^

**Figure 3:**
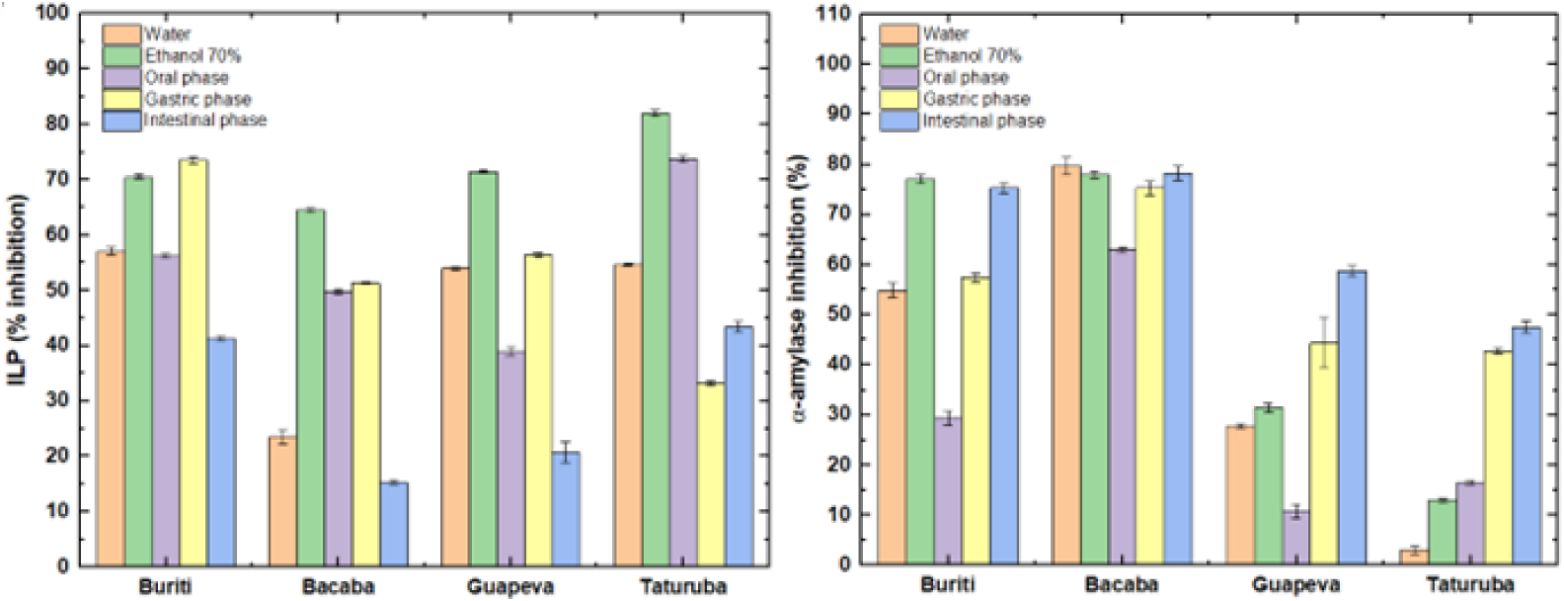
*In vitro* biological properties of buriti, bacaba, guapeva, and taturubá pulp before and after the *in vitro* digestion phases (oral, gastric, and intestinal).

### 3.6 *In Vitro* Biological Activities during Simulation of *In Vitro* Gastrointestinal Digestion

The analysis of Figure 3 reveals that the undigested (ethanolic) extract of bacaba, guapeva, and taturubá pulps demonstrated the greatest inhibition of lipid peroxidation in the biological system (egg yolk), with values of 64.49%, 71.45%, and 82.05%, respectively. On the other hand, buriti pulp exhibited the highest level of inhibition in the gastric phase (73.53%). In general, the greatest significant reductions in antioxidant capacity were observed between the gastric and intestinal phases for all fruits analyzed (*p* < 0.05). This decrease between the digestive phases is consistent with the findings of Chen et al.^67^ and Schulz et al.,^61^ which suggest that the digestive process can negatively impact the antioxidant potential, mainly due to the reduction in the content of phenolic compounds and their transformation into altered structural forms with distinct chemical properties. Furthermore, factors such as variations in pH, particle size, and hydrolysis of phenolics during digestion may contribute to this decrease in antioxidant capacity observed in the different phases of the digestive process.

The enzyme α-amylase is responsible for the breakdown of carbohydrates. Inhibition of this enzyme, which is the first enzyme required in starch hydrolysis, slows the absorption of monosaccharides and reduces the rate of glucose release into the blood, which is recognized as an important strategy for the treatment of postprandial hyperglycemia.^68^ Figure 3 also shows the results of α-amylase enzyme inhibition before, during, and after the simulated *in vitro* gastrointestinal digestion process. Notably, the intestinal phase showed the highest levels of inhibition (72.24, 78.23, 58.65, and 47.53%) for buriti, bacaba, guapeva, and taturubá, respectively. A similar result was reported in a previous study, in which the α-amylase inhibitory effects of 22 fruit juices were almost all enhanced after simulating *in vitro* gastrointestinal digestion.^69^ However, some inconsistent results were also reported. Burgos-Edwards et al.^70^ reported that the α-amylase inhibitory activities of two wild Chilean gooseberries (*Ribes magellanicum* and *Ribes punctatum*) decreased after gastric digestion but recovered significantly after intestinal digestion. These differences may be due to the different compositions of the phytochemical components. In addition to phenolic compounds, other substances released during digestion may also exert α-amylase inhibitory activity. Previous studies have found that the polysaccharide digestive products in quinoa (*Chenopodium quinoa*) fruit juice and protein exhibited a good inhibitory effect on α-amylase after simulated gastrointestinal digestion.^69,71^ This study found that the pulp of the analyzed fruits, after all stages of digestion, effectively inhibited α-amylase.

## 4 Conclusions

The results of this study show that *in vitro* gastrointestinal digestion significantly affects the phenolic composition and antioxidant potential of the pulps of native Brazilian fruits such as bacaba, guapeva, taturubá, and buriti. Although there was a general reduction in the concentrations of phenolic compounds after digestion, some fruits presented interesting variations, such as the increase in the antioxidant capacity of guapeva in the intestinal phase, suggesting transformations or the release of additional bioactive compounds. In addition, the study highlighted the crucial impact of the choice of extraction solvent, with notable differences in the bioaccessibility of the compounds depending on the affinity of the solvents and the specific characteristics of the food matrix, with water being the solvent that presented the highest percentage of bioaccessibility. The therapeutic potential of these fruits reinforces the relevance of considering the bioaccessibility of phenolic compounds for the development of functional foods. Thus, this study contributes to understanding bioactive compounds’ dynamics during digestion and their implications for human health, pointing out new opportunities for applying these fruits in areas such as nutrition and pharmacology. Future research should focus on *in vivo* validation, including studies with larger sample sizes, assessment of bioavailability in biological systems, and identification of metabolites formed during digestion and metabolism. These steps will be essential to confirm the physiological relevance and potential health-promoting properties suggested by our *in vitro* findings.

## Supplementary Material



## Notes

Supplementary Note 1: Calibration curves of antioxidant potential protocols (FRAP, CUPRAC, TRC) (PDF)
