# Supplementary material for: Application of the INFOGEST Protocol to Evaluate the Effects of Simulated In Vitro Gastrointestinal Digestion on Polyphenols and Antioxidant Potential of Four Native Amazonian Fruits
Source: ACS Omega. 2025 Nov 20;10(48):59872–84. doi: 10.1021/acsomega.5c09934 (PMC12772399; doi:10.1021/acsomega.5c09934)
Supplement: Supplementary file 1 [file ao5c09934_si_001.pdf]

Supplementary Material

Application of the INFOGEST protocol to evaluate the effects of simulated *in vitro* gastrointestinal digestion on polyphenols and antioxidant potential of four native Amazonian fruits

Rômulo Alves MORAIS [1]*, Hermanny Matos Silva SOUSA [2], Glêndara Aparecida de Souza MARTINS [1]

[1] *Graduate Program in Food Science and Technology, Department of Food Science and Technology, Federal University of Tocantins (UFT), Palmas 77001-090, Brazil.*
[2] *Department of Food Science, Federal University of Lavras (UFLA), Lavras, 37200-000, Brazil.*

* Corresponding author: Romulo Alves Morais (MORAIS, R. A.) (romuloitallo2505@gmail.com). Department of Food Science and Technology, Federal University of Tocantins (UFT), Palmas 77001-090, Brazil.

FRAP				
Ascorbic acid equivalent				
**n**	**C (mg/L)**	**ABS1**	**ABS2**	**Average**
1	30	0,89	0,845	0,868
2	60	1,444	1,095	1,054
7	210	2,626	1,933	1,981
8	240	2,758	2,204	2,251
9	270	2,899	2,48	2,450
10	300	3,089	2,67	2,685

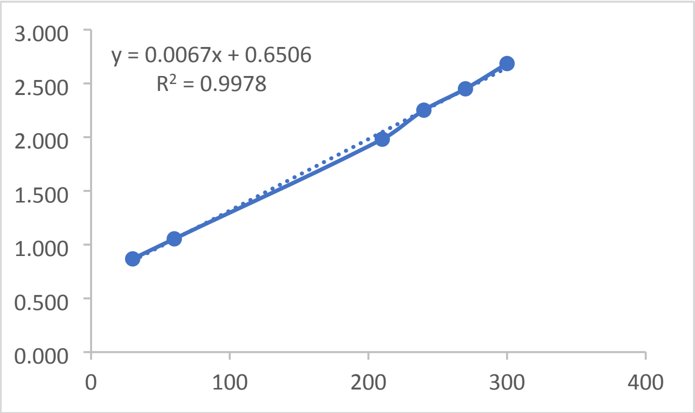

Figure S1. Calibration curve for the FRAP protocol.

n	Trolox (u mol/L)	ABS1	ABS2	Average
1	6000	3,086	3,076	3,081
2	4000	2,012	2,014	2,013
3	3000	1,445	1,445	1,445
6	1500	0,68	0,692	0,686
8	500	0,158	0,157	0,1575
9	375	0,115	0,112	0,1135

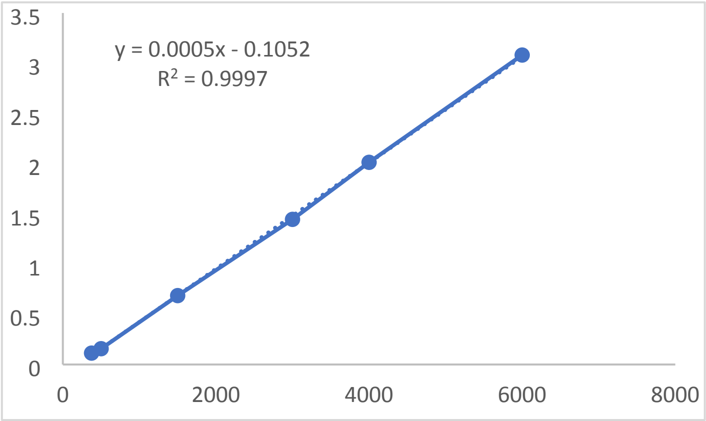

Figure S2. Calibration curve for the CUPRAC protocol.

Reduction Capacity Curve				
Quercetin				
**n**	**C (mg/L)**	**ABS1**	**ABS2**	**Abs Average**
1	720	1,355	1,398	1,3765
3	500	1,095	1,077	1,086
4	252	0,708	0,699	0,7035
6	72	0,372	0,37	0,371
8	50	0,257	0,27	0,2635
9	25	0,18	0,197	0,1885
10	0	0,15	0,166	0,158

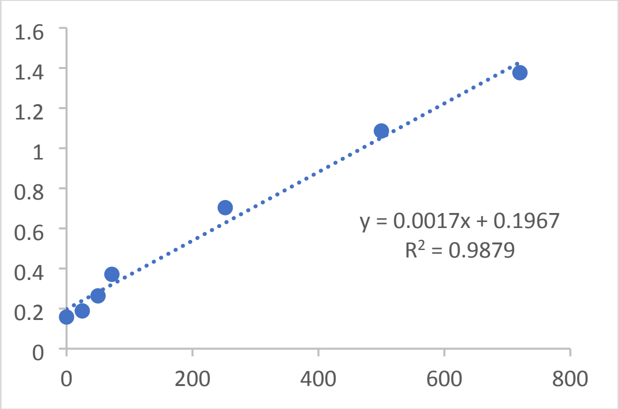

Figure S3. Calibration curve for the TRC protocol.
